# Autistic Trait Profiles Across Mood and Psychotic Spectrum Disorders: A Transdiagnostic Outpatient Study

**DOI:** 10.3390/jcm15124659

**Published:** 2026-06-16

**Authors:** Michele Ribolsi, Antonio Maria D’Onofrio, Alexia Koukopoulos, Federico Fiori Nastro, Martina Pelle, Alessandro Michele Giannico, Sara Barbonetti, Lodovico Maria Balzoni, Marco Cataldo Zaza, Giorgio Di Lorenzo, Gabriele Sani, Giovanni Camardese

**Affiliations:** 1 Department of Life Science, Health, and Health Professions, Link Campus University, 00165 Rome, Italy; 2 Department of Neuroscience, Section of Psychiatry, Università Cattolica del Sacro Cuore, 00168 Rome, Italy; 3 Department of Systems Medicine, Tor Vergata University of Rome, 00133 Rome, Italy; 4 IRCCS Fondazione Santa Lucia, 00179 Rome, Italy; 5 Unit of Neurology, Neurophysiology, Neurobiology and Psychiatry, Department of Medicine, Campus Bio-Medico University, 00128 Rome, Italy; 6 Università Cattolica del Sacro Cuore, 00168 Rome, Italy; 7 Department of Neuroscience, Head-Neck and Chest, Section of Psychiatry, Fondazione Policlinico Universitario Agostino Gemelli IRCCS, 00168 Rome, Italy

**Keywords:** autistic traits, psychotic spectrum disorder, bipolar disorder, major depressive disorder, social cognition, PANSS Autism Severity Score, psychiatric comorbidity, dimensional psychiatry, personalized treatment

## Abstract

**Background/Objectives**: Autistic traits are distributed dimensionally across psychiatric populations, yet their systematic assessment in mood and psychotic spectrum disorders remains limited. While elevated autistic traits have been documented in schizophrenia spectrum disorders, evidence in bipolar disorder (BD) and major depressive disorder (MDD) is scarce, and no studies have applied the clinician-rated PANSS Autism Severity Score (PAUSS) to mood disorder populations. This study aims to investigate the presence and severity of autistic traits across psychotic spectrum disorder (PSD), BD, and MDD in an outpatient sample using the PAUSS. **Methods**: In this cross-sectional naturalistic outpatient study, clinically stable adult patients with MDD, BD, or PSD, without autism spectrum disorder, were assessed with the Brief Psychiatric Rating Scale (BPRS) and PAUSS. Group comparisons, adjusted models, correlation analyses, principal component analysis, and multinomial logistic regression were performed. **Results**: A total of 165 patients were included (MDD, *n* = 84, BD, *n* = 45, PSD, *n* = 36). Compared with the mood disorder groups, PSD patients were younger and showed higher BPRS scores. PSD was also characterized by significantly higher PAUSS total, social, and communication scores, whereas PAUSS RRB did not differ in univariate analyses. In the overall sample, BPRS severity correlated positively with all PAUSS dimensions, while age showed only weak or non-significant associations. Diagnosis-stratified analyses revealed that the association between psychopathology and autistic traits was present in MDD and BD, but not in PSD. PCA showed that autistic trait dimensions converged on a broad common profile and differed across diagnostic groups, with PSD showing the most distinct pattern. In multinomial logistic regression, higher BPRS, higher PAUSS social and communication scores, and younger age independently distinguished PSD from MDD and BD; PAUSS RRB showed an inverse association only in the multivariable model. **Conclusions**: This study supports a transdiagnostic perspective on autistic traits in adult psychiatric populations, highlighting disorder-specific differences across diagnostic categories. Social and communication impairments emerged as key dimensions distinguishing PSD from mood disorders. Assessing autistic traits in psychiatric settings may improve diagnostic precision and inform personalized, stratified treatment approaches.

## 1. Introduction

Autism Spectrum Disorder (ASD) is a complex neurodevelopmental condition characterized by persistent deficits in social communication and interaction, restricted and repetitive patterns of behavior, and clinically significant impairment in daily functioning [1]. Conceptualized as a spectrum encompassing a wide range of neurodevelopmental profiles [2,3], ASD affects approximately 1 in 127 individuals worldwide, over 61.8 million people [4], with an adult prevalence of 2.21% in the United States and a male-to-female ratio of 3.8:1 [5]. This population, projected to exceed 75 million globally by 2030 [6], faces elevated risks of psychiatric comorbidities and unemployment rates up to 85% [7,8,9].

ASD rarely occurs in isolation: up to 70% of autistic individuals meet criteria for at least one additional diagnosis [10]. Among the most prevalent comorbidities, Attention-Deficit/Hyperactivity Disorder (ADHD) affects 30–50% [11,12], anxiety disorders 9.4–42% [7,8], depression 23–48.6% [13,14], and Obsessive–Compulsive Disorder (OCD) 17–37% [15,16]. Eating disorders, particularly anorexia nervosa, are overrepresented among autistic females [17], alongside psychotic spectrum disorder (PSD) (6–37.5%) [18,19], bipolar disorder (7–21%) [20,21], and elevated rates of suicidal ideation and attempts [22]. Although historically conceptualized as closely related [23], ASD and schizophrenia are now recognized as distinct entities [24,25], with growing research renewing interest in their overlap and comorbidity [26,27,28,29]. Similarly, BD occurs at substantially higher rates in autistic adults, with pooled prevalence estimates of 5–7.5% [30,31] and has been described as a distinct clinical phenotype characterized by atypical mood presentations, emotional dysregulation, and reduced tolerability to antipsychotic treatment [32].

Autistic traits are subclinical characteristics encompassing behavioral, cognitive, and personality features distributed across both clinical and non-clinical populations [33], and have been linked to interpersonal difficulties, impairments in social attention, and limited social skills [34,35,36]. Seminal work by Baron-Cohen et al. (2001) [37] demonstrated that ASD-related features are continuously distributed in the general population, differing in degree rather than in kind [38,39,40], supporting the development of quantitative assessment tools including the Autism-Spectrum Quotient (AQ) [37], the Social Responsiveness Scale (SRS) [41], the Broad Autism Phenotype Questionnaire (BAPQ) [42], and the Ritvo Autism Asperger Diagnostic Scale-Revised (RAADS-R) [43].

In PSD, 20–50% of patients score above ASD thresholds on such measures, with autistic traits correlating with negative symptoms and social withdrawal [44], while findings in clinical-high risk for psychosis (CHR-P) populations remain inconclusive [45,46,47]. In BD, despite historical underrepresentation in clinical trials [48], emerging evidence indicates elevated autistic traits in 42.7–47% of patients [49,50], associated with earlier illness onset, longer hospitalizations, and higher rates of anxiety disorders.

The PANSS Autism Severity Score (PAUSS) [51], a clinician-rated instrument derived from 12 Positive and Negative Syndrome Scale (PANSS) [52] items capturing social withdrawal, impaired rapport, stereotyped thinking, and reduced social engagement, has demonstrated good internal consistency and convergent validity with Autism Diagnostic Observation Schedule (ADOS) and Autism Diagnostic Interview-Revised (ADI-R) diagnoses, outperforming self-report measures in psychotic populations. Studies using the PAUSS identified elevated autistic traits in 20–50% of individuals with PSD, with higher scores associated with poorer neurocognitive performance, greater negative symptom burden, and reduced global functioning [53]. To date, however, no studies have extensively applied the PAUSS to mood disorder populations. The present study, therefore, aims to investigate the presence and severity of autistic traits in an outpatient sample of individuals with PSD compared to those with bipolar disorder (BD) and major depressive disorder (MDD), using the PAUSS, to better characterize autistic phenotypes beyond acute symptom states and address the limitations of prior studies relying on self-report tools or inpatient samples.

## 2. Materials and Methods

### 2.1. Study Design

This study was a cross-sectional, observational, naturalistic investigation conducted in an outpatient psychiatric setting, aimed at characterizing autistic traits across psychiatric diagnostic groups in a real-world clinical context. Participants were consecutively recruited during routine ambulatory psychiatric care, without any experimental allocation or protocol-driven modification of treatment, so that enrollment reflected ordinary clinical practice rather than a preselected research sample.

### 2.2. Participants

The sample included adult outpatients with a clinical diagnosis of MDD, BD, or PSD, established by the treating psychiatrist using standard diagnostic assessment procedures in routine care. The PSD group included patients with schizophrenia spectrum and other psychotic disorders according to DSM-5 criteria, specifically schizophrenia, schizoaffective disorder, and brief psychotic disorder. In order to reduce the potential confounding effect of acute psychopathological disorganization on both symptom ratings and autistic trait assessment, only patients who were judged to be in a condition of relative psychopathological compensation/stability at the time of evaluation were considered eligible, whereas subjects in an acute phase, including marked affective, psychotic, or behavioral decompensation requiring urgent/intensive management, were excluded. Additional exclusion criteria comprised a lifetime diagnosis of autism spectrum disorder, severe cognitive impairment, neurological or clinical conditions precluding reliable psychopathological assessment, and inability or unwillingness to provide informed consent. The explicit exclusion of subjects with a diagnosis of autism spectrum disorder was intended to ensure that the study addressed the distribution and clinical correlates of autistic traits within psychiatric populations not formally diagnosed with autism, rather than the overlap between psychiatric disorders and established neurodevelopmental conditions. Because the study followed a naturalistic design, psychopharmacological treatment was not standardized by protocol and was not manipulated for research purposes; instead, each patient continued the treatment prescribed by the psychiatrist routinely in charge of clinical care, according to the standard of care, including, when clinically indicated, antidepressants, mood stabilizers, and antipsychotics, either alone or in combination. Given the observational, exploratory, and naturalistic nature of the study, and the fact that the sample size was determined by the number of eligible patients available during the recruitment period rather than by experimental allocation, no formal a priori power analysis was performed.

### 2.3. Psychometric Assessment

Psychopathological assessment was based on clinician-administered instruments, specifically the Brief Psychiatric Rating Scale (BPRS), used as a measure of global psychopathology severity [54], and the PANSS Autism Severity Score (PAUSS), used to quantify autistic traits both as a total score and across its principal dimensions [55]. These scales were administered in the outpatient setting by trained clinicians as part of the study procedure. The use of a rater-administered instrument, rather than a self-report questionnaire, was intended to minimize potential subjective overestimation and to ensure a more clinically informed evaluation of autistic traits.

### 2.4. Ethical Considerations

The study was conducted in accordance with the principles of the Declaration of Helsinki and with local regulatory and ethical standards for observational clinical research; the study was approved by the Independent Ethics Committee of Policlinico Tor Vergata (#184/25; 26 June 2025) and all participants provided written informed consent prior to inclusion.

### 2.5. Statistical Analysis

Descriptive statistics were reported as mean ± SD for normally distributed variables and median (IQR) for non-normally distributed variables. Normality was assessed using the Shapiro–Wilk test within each diagnostic group. Raw between-group differences were tested using one-way ANOVA or Kruskal–Wallis tests, as appropriate, followed by Tukey or Dunn-Bonferroni post hoc comparisons. To account for potential confounding, all outcomes were additionally examined in age-adjusted linear regression models. Because general psychopathology may influence autistic trait expression, PAUSS total and subdomain scores were further analyzed in models adjusted for both age and BPRS total score, whereas BPRS was not adjusted for itself. Adjusted pairwise contrasts were estimated using estimated marginal means with Bonferroni correction.

Correlation analyses were conducted to assess the relationships between age, BPRS total score, and PAUSS total and subdomain scores. Owing to the non-normal distribution of several variables, associations in the overall sample were estimated using Spearman’s rank correlation coefficient (ρ). Additional diagnosis-stratified correlation analyses were then performed within each group using Pearson’s or Spearman’s coefficients, as appropriate according to subgroup-specific distributional assumptions.

To investigate the multivariate structure of autistic trait dimensions, a principal component analysis (PCA) was performed on the PAUSS social, communication, and restricted/repetitive behavior (RRB) subdomain scores using a correlation-based decomposition. The proportion of variance explained by each component was inspected through eigenvalues and scree plot visualization, and component loadings were used to interpret the contribution of each PAUSS dimension to the latent structure. To test whether the multivariate PAUSS profile differed across diagnostic groups, a permutational multivariate analysis of variance (PERMANOVA) was conducted on the PAUSS matrix using 999 permutations.

Finally, to evaluate the independent contribution of demographic, clinical, and autistic trait variables to diagnostic group membership, a multinomial logistic regression model was fitted with diagnosis as the dependent variable and age, gender, BPRS total score, and PAUSS subdomain scores as predictors. Results were expressed as odds ratios (ORs) with 95% confidence intervals (95% CIs). Model performance was assessed by comparing the fitted model with the null model using a likelihood ratio test, while explanatory power was summarized using McFadden’s pseudo-R^2^. Multicollinearity was examined using the variance inflation factor (VIF), and predictive performance was summarized descriptively as classification accuracy from the confusion matrix.

All analyses were performed in RStudio, version 2024.12. Statistical significance was set at *p* < 0.05, and all tests were two-tailed.

## 3. Results

### 3.1. Diagnostic Group Differences in Psychopathology and Autistic Traits

A total of 165 participants were included in the study: 84 with MDD, 45 with BD, and 36 with PSD.

The three diagnostic groups differed significantly in age (Kruskal–Wallis χ^2^ = 11.59, df = 2, *p* = 0.003). Median age was 48 years [IQR 30.75–60] in MDD, 50 years [34.5–58.5] in BD, and 36 years [28.75–46.25] in PSD. Post hoc comparisons showed that the PSD group was significantly younger than both BD (*p* = 0.007) and MDD (*p* = 0.006), whereas MDD and BD did not differ in age (*p* = 1.00). By contrast, gender distribution did not differ across groups (χ^2^ = 3.95, df = 2, *p* = 0.139). The proportion of females was 63.9% in MDD, 60.0% in BD, and 44.4% in PSD.

Across groups, BPRS scores differed significantly, with the PSD group showing greater overall psychopathology than both MDD (*p* < 0.001) and BD (*p* < 0.001), whereas no difference emerged between MDD and BD; this pattern remained unchanged after adjustment for age. Regarding autistic traits, PAUSS total, PAUSS social, and PAUSS communication scores were all significantly higher in PSD than in both MDD (all *p* < 0.001) and BD (all *p* < 0.001), while MDD and BD did not differ from each other. These findings were observed in the raw analyses, persisted after adjustment for age, and remained significant after further adjustment for BPRS, indicating that group differences in these PAUSS dimensions were not fully explained by age or by general psychopathology severity. By contrast, PAUSS RRB scores did not differ significantly across groups in raw, age-adjusted, or age- plus BPRS-adjusted analyses. Overall, the results indicate that the PSD group is characterized by greater social-communication autistic traits, but not by increased restricted and repetitive behaviors, relative to MDD and BD. Full statistical details are reported in Table 1.

### 3.2. Correlation Analyses

Spearman correlation analyses showed no significant associations between age and most PAUSS dimensions, except for a weak negative correlation with PAUSS communication (ρ = −0.157, *p* = 0.045). Associations between age and PAUSS total (ρ = −0.146, *p* = 0.062) and PAUSS social (ρ = −0.148, *p* = 0.058) showed only trend-level significance, whereas PAUSS RRB was not associated with age (ρ = −0.034, *p* = 0.669). In contrast, psychopathology severity (BPRS) was strongly and positively associated with all PAUSS dimensions. Higher BPRS scores correlated with PAUSS total (ρ = 0.557, *p* < 0.001), PAUSS social (ρ = 0.500, *p* < 0.001), PAUSS communication (ρ = 0.536, *p* < 0.001), and PAUSS RRB (ρ = 0.340, *p* < 0.001).

**Figure 1:**
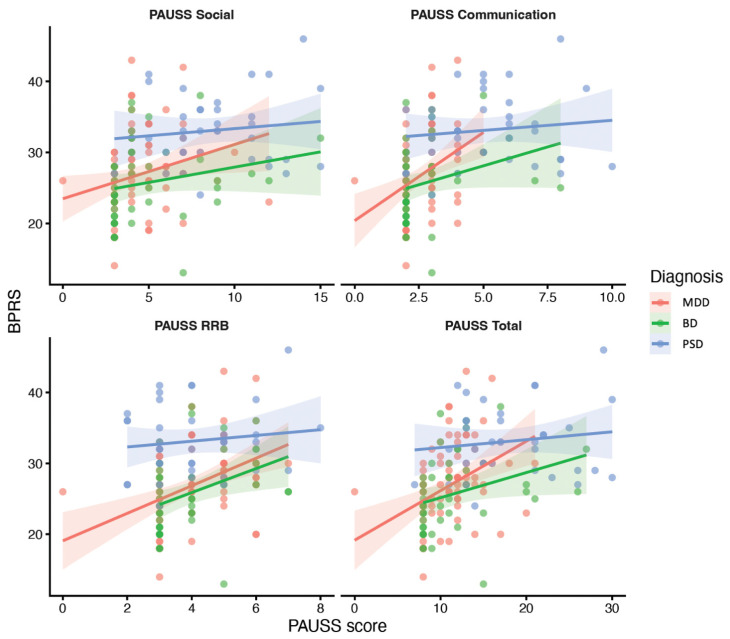
Diagnosis-stratified associations between psychopathology severity and autistic traits. Scatterplots showing the relationship between BPRS scores and PAUSS social, communication, restricted, and repetitive behaviors (RRB), and total scores across the three diagnostic groups (MDD, BD, and PSD). Solid lines represent group-specific linear fits, with shaded areas indicating 95% confidence intervals. Consistent positive associations between psychopathology severity and autistic trait dimensions were observed in MDD and BD, whereas these relationships were attenuated and non-significant in PSD. Abbreviations: BD, bipolar disorder; BPRS, Brief Psychiatric Rating Scale; MDD, major depressive disorder; PAUSS, PANSS Autism Severity Score; PSD, psychotic spectrum disorder; RRB, restricted and repetitive behaviors.

Given the significant associations observed in the overall sample, correlation analyses between BPRS scores and PAUSS dimensions were further examined within each diagnostic group. In MDD, BPRS scores showed moderate positive correlations with all PAUSS dimensions, including PAUSS social (r = 0.402, *p* = 0.0002), PAUSS communication (r = 0.437, *p* < 0.001), PAUSS RRB (r = 0.473, *p* < 0.001), and PAUSS total (r = 0.498, *p* < 0.001). Similarly, in BD, BPRS severity was positively associated with all PAUSS domains, including PAUSS social (r = 0.403, *p* = 0.006), PAUSS communication (r = 0.387, *p* = 0.009), PAUSS RRB (r = 0.439, *p* = 0.003), and PAUSS total (r = 0.475, *p* = 0.001). In contrast, no significant correlations emerged in the PSD group, where associations between BPRS and PAUSS dimensions were weak and non-significant (all *p* > 0.44). These relationships are visually illustrated in Figure 1, which displays the scatterplots and regression lines for each diagnostic group.

### 3.3. Principal Component Analysis

**Figure 2:**
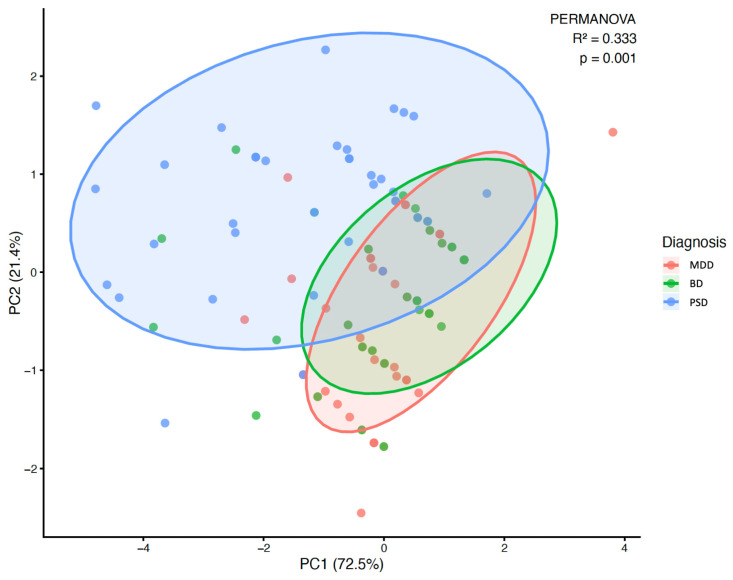
Principal component analysis of PAUSS dimensions across diagnostic groups. PCA was conducted using PAUSS social, communication, and restricted/repetitive behavior (RRB) scores. PC1 explained 72.5% of the total variance and reflected a broad autistic trait dimension, whereas PC2 explained 21.4% and was mainly driven by the RRB domain. Ellipses represent the group-wise distribution in PCA space. The multivariate structure differed significantly across diagnoses (PERMANOVA: R^2^ = 0.333, *p* = 0.001), with greater separation of the PSD group relative to MDD and BD. Abbreviations: BD, bipolar disorder; MDD, major depressive disorder; PC, principal component; PSD, psychotic spectrum disorder.

A principal component analysis (PCA) was performed on the three PAUSS subdomains (social, communication, and restricted/repetitive behaviors [RRB]) to examine their joint multivariate structure. The first principal component (PC1) accounted for 72.5% of the total variance, whereas PC2 and PC3 explained 21.4% and 6.1%, respectively, yielding a cumulative explained variance of 93.9% for the first two components. The loading structure indicated that PC1 captured a general autistic trait dimension, with substantial contributions from PAUSS social (loading = −0.611), PAUSS communication (loading = −0.625), and PAUSS RRB (loading = −0.485). In contrast, PC2 was primarily driven by the RRB dimension (loading = −0.872), suggesting that repetitive/restricted behaviors represent a partially distinct source of variance relative to the social-communication dimensions. To test whether the multivariate PAUSS profile differed across diagnostic categories, a PERMANOVA was conducted on the PAUSS matrix. This analysis showed a significant effect of diagnosis (F = 40.41, R^2^ = 0.333, *p* = 0.001), indicating that diagnostic group membership explained approximately one-third of the multivariate variance in PAUSS dimensions. As illustrated in Figure 2, the PCA projection showed a clearer separation of the PSD group from the mood disorder groups, whereas MDD and BD displayed greater overlap.

### 3.4. Multinomial Logistic Regression

A multinomial logistic regression model was fitted to test whether age, gender, BPRS, and PAUSS subdomain scores independently discriminated among diagnostic groups. The overall model significantly improved fit relative to the null model (likelihood ratio χ^2^ = 114.94, df = 12, *p* < 0.001), with a McFadden pseudo-R^2^ of 0.342 and an overall classification accuracy of 66.9%. Multicollinearity was low (all VIFs < 1.6).

**Figure 3:**
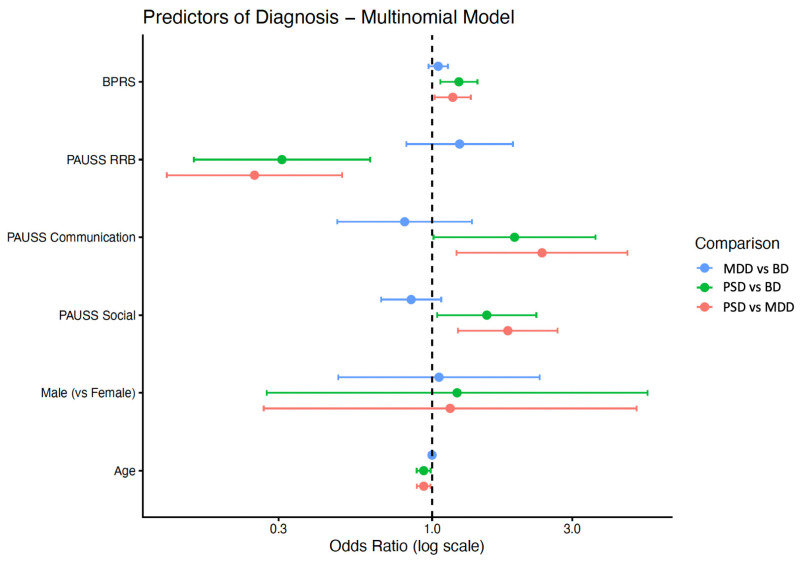
Forest plot of multinomial logistic regression estimates for diagnostic group comparisons. Odds ratios (ORs) and 95% confidence intervals from the multinomial logistic regression model examining the association of age, gender, BPRS, and PAUSS subdomains with diagnostic group membership. Comparisons are shown for MDD vs. BD, PSD vs. BD, and PSD vs. MDD. The dashed vertical line indicates OR = 1 (no association). Positive discrimination of PSD relative to mood disorder groups was primarily driven by higher BPRS, higher PAUSS social and communication scores, and lower PAUSS RRB scores, whereas gender was not a significant predictor. Age also significantly contributed, indicating that PSD participants were younger than both BD and MDD patients. Abbreviations: BD, bipolar disorder; BPRS, Brief Psychiatric Rating Scale; MDD, major depressive disorder; PAUSS, PANSS Autism Severity Score; PSD, psychotic spectrum disorder; RRB, restricted and repetitive behaviors.

In adjusted comparisons, BPRS remained significantly higher in PSD than in both BD (OR = 1.23, 95% CI 1.07–1.43, *p* = 0.005) and MDD (OR = 1.18, 95% CI 1.02–1.35, *p* = 0.024), whereas no difference emerged between BD and MDD. PAUSS communication significantly distinguished PSD from BD (OR = 1.91, 95% CI 1.01–3.60, *p* = 0.046) and PSD from MDD (OR = 2.37, 95% CI 1.21–4.63, *p* = 0.012). Similarly, PAUSS social differentiation distinguished PSD from BD (OR = 1.53, 95% CI 1.04–2.27, *p* = 0.031) and from MDD (OR = 1.81, 95% CI 1.22–2.68, *p* = 0.003). By contrast, PAUSS RRB showed an inverse association, being lower in PSD than in both BD (OR = 0.31, 95% CI 0.15–0.61, *p* < 0.001) and MDD (OR = 0.25, 95% CI 0.12–0.49, *p* < 0.001). Age, but not gender, also contributed to differentiating PSD from the mood disorder groups. As shown in Figure 3 (forest plot), the multivariable pattern was therefore characterized by higher psychopathology severity and greater social-communication autistic traits in PSD, together with lower RRB scores.

Interestingly, although PAUSS RRB did not significantly differ across groups in the univariate analyses, it emerged as a significant discriminator in the multinomial model, indicating that its effect became apparent only after accounting for shared variance with age, gender, BPRS, and the other PAUSS dimensions. To further clarify this suppression effect, we fitted a series of nested multinomial logistic regression models, sequentially introducing age, sex, BPRS, and the remaining PAUSS subscales as covariates (Supplementary Table S1). PAUSS RRB did not discriminate PSD from MDD in unadjusted analyses (OR = 1.04, 95% CI 0.77–1.41, *p* = 0.793), and the addition of age and sex produced no meaningful change in this estimate. The inclusion of BPRS was associated with a directional shift in the OR for PSD vs. MDD (OR = 0.77, 95% CI 0.53–1.11, *p* = 0.156), which however did not reach statistical significance. The decisive change occurred upon the simultaneous entry of PAUSS social and PAUSS communication scores, which reduced the OR to 0.25 (95% CI 0.12–0.49, *p* < 0.001). These results indicate that the primary suppressor is the shared variance between PAUSS RRB and the social-communication subscales, rather than age, sex, or general psychopathology severity.

Age significantly differentiated PSD from both BD (OR = 0.94, 95% CI 0.89–0.99, *p* = 0.019) and MDD (OR = 0.94, 95% CI 0.89–0.99, *p* = 0.017), indicating that PSD participants were younger than patients in the two mood disorder groups. Gender did not significantly differentiate any diagnostic comparison.

## 4. Discussion

The present study compared the presence of autistic traits across three clinical groups, MDD, BD, and PSD, providing further insight into the transdiagnostic and disorder-specific features of autism-related dimensions in adult psychiatric populations. Moreover, the correlation between the levels of autistic traits and the degree of psychopathological severity was investigated.

Consistent with prior evidence, patients with PSD exhibited greater overall psychopathological severity, as reflected by higher BPRS scores, compared to those with mood disorders [56,57,58]. This finding aligns with the well-established clinical profile of psychotic disorders, which are often characterized by earlier onset and more severe symptomatology [59,60].

In addition, PSD patients demonstrated significantly higher levels of autistic traits, particularly in the social and communication domains [61]. These results are consistent with previous literature [44,46] and support the hypothesis that social cognition and interpersonal functioning deficits represent a key area of overlap between psychotic disorders and autism-related phenotypes [62]. In contrast, RRB did not differ significantly in univariate analyses, suggesting that this dimension may be less discriminative across diagnostic categories or less prominently expressed in adult psychiatric samples [63,64].

A relevant clinical limitation concerns the overlap between negative symptoms of psychosis and autistic features related to social withdrawal and interpersonal functioning. Differentiating schizophrenia negative symptoms from autistic symptoms represents a pivotal but complicated task even for expert psychiatrists [65,66]. In particular, reduced social engagement, diminished spontaneous communication, and apparent interpersonal detachment may be observed in both conditions, although they may arise from partially different underlying mechanisms. From a clinical perspective, these dimensions can be difficult to disentangle, as they often converge in similar behavioral manifestations. This overlap highlights the need for cautious interpretation of measures capturing social isolation and supports the importance of integrating standardized assessments with a detailed clinical and developmental evaluation.

Across the overall sample, a significant positive association was observed between psychopathological severity and all dimensions of autistic traits. This finding further supports the conceptualization of autistic traits as dimensional constructs that systematically co-vary with overall symptom burden, rather than being confined to categorical diagnostic frameworks. In line with this perspective, a study conducted on a sample of 263 adolescent outpatients reported that higher levels of autistic traits were significantly associated with increased symptomatology across multiple domains, including mood and anxiety disturbances, eating disorder severity, psychotic symptoms, and personality features such as detachment and vulnerable narcissism [67]. However, diagnosis-stratified analyses revealed a more nuanced picture. While significant associations between BPRS scores and autistic traits were observed in MDD and BD, this relationship was not evident in PSD. This finding is partially consistent with the study by Matsuo et al. (2015) [44], which reported that a substantial proportion of adults with bipolar disorder and schizophrenia exhibited high autistic-like traits or symptoms, independent of overall symptom severity. One possible interpretation is that, within mood disorders, autistic traits may be more state-dependent and fluctuate with clinical severity. Conversely, in PSD, these traits may reflect more stable, trait-like characteristics, potentially linked to neurodevelopmental vulnerability, and therefore less sensitive to changes in current psychopathology. This distinction may point to different underlying mechanisms and temporal dynamics of autistic features across psychiatric conditions.

The PCA shows a clear latent structure among the PAUSS subdomains. The first principal component accounted for most of the variance, with comparable loadings from social, communication, and RRB domains, supporting its interpretation as a general autistic trait factor. The second principal component was primarily driven by RRB, indicating that restricted and repetitive behaviors represent a partially distinct dimension relative to social-communication features. The PCA projection showed a clearer separation of the psychosis spectrum disorder group from the mood disorder groups, whereas major depressive disorder and bipolar disorder displayed greater overlap. This finding is consistent with emerging models proposing partial overlap between autism and psychosis spectrum, alongside disorder-specific alterations in social cognition and behavioral organization [68].

Furthermore, we used multinomial logistic regression to clarify the independent contributions of key variables. Higher psychopathological severity, greater impairments in social interaction and communication, and younger age significantly increased the likelihood of belonging to the PSD group relative to MDD and BD.

Several limitations of this study should be acknowledged. First, the cross-sectional design precludes any inference about causality or the temporal stability of the observed associations.

A recent longitudinal study by Pelizza et al. [53] reported poor long-term stability of PAUSS in individuals with first-episode psychosis (FEP), showing a significant decrease over time. However, FEP populations might present heterogeneous and unstable clinical trajectories, particularly in terms of negative and disorganized symptoms, which may have influenced changes in PAUSS scores across follow-up assessments. However, we are not aware of studies specifically investigating the long-term stability of autistic traits in clinical populations with mood disorders. Future longitudinal follow-up of our sample will be necessary to examine whether, and to what extent, the observed results remain stable over time. Second, the sample size, although adequate for exploratory analyses, may limit the generalizability of the findings and the statistical power of subgroup analyses. In particular, the BD sample consisted mostly of BD type II. Third, the relatively small size of the PSD subgroup (n = 36) warrants caution in interpreting the multinomial logistic regression results. Multinomial regression with multiple simultaneous predictors requires adequate observations per group to produce stable and reliable estimates, and the risk of overfitting increases as the number of predictors approaches the effective sample size of the smallest class. Similarly, subgroup-specific correlation analyses conducted within the PSD group should be interpreted with particular caution, given the limited statistical power to detect associations of small-to-moderate magnitude. Future studies should replicate these findings in larger, independently recruited PSD samples. Fourth, the assessment of autistic traits relied on the PAUSS, which, while validated in psychiatric populations, is derived from a general psychopathology scale and may not capture the full complexity of autism spectrum features compared to gold-standard diagnostic instruments, such as the ADOS-2 [69,70]. Since the PAUSS was originally developed and validated in schizophrenia spectrum disorders, its application in mood disorder populations should be interpreted with caution. Some PANSS-derived dimensions, particularly negative symptoms, may overlap with depressive symptomatology and could lead to an overestimation of autistic traits rather than reflecting genuine autism spectrum features, although the euthymic state of our sample limits this confounder. The construct validity of the PAUSS in mood disorders nonetheless remains insufficiently established. Our dataset did not include autism-specific measures, such as the Autism-Spectrum Quotient (AQ), precluding a direct assessment of convergent validity. Future studies should systematically compare PAUSS scores with the AQ and other validated autism-specific tools to clarify the specificity of the construct in mood disorder populations. Furthermore, while the PAUSS shows good overall internal consistency in mixed psychiatric samples, recent psychometric work has highlighted that specific items (particularly G5 and G15) may exhibit suboptimal inter-item correlations in some populations, and the long-term stability of individual PAUSS items has been questioned in longitudinal studies of psychosis [53]. These observations suggest that ongoing refinement of the scale’s item-level structure remains a relevant direction for future psychometric research; however, they do not undermine the use of the PAUSS total and subdomain scores as dimensional indicators of autistic traits in the present study. It is also worth noting that the use of the PAUSS offers several advantages over self-report measures for assessing autistic traits, particularly in clinical populations [24,71].

The PAUSS scale showed a good convergence with the ADOS diagnosis, whereas other self-report instruments (such as the Autism Questionnaire and the Empathy Quotient) did not show similar characteristics [71]. As an observer-rated instrument derived from clinician-administered assessments, the PAUSS is less susceptible to biases related to insight, self-awareness, and social desirability, which can affect self-report data. This is especially relevant in individuals with impaired introspection or communication difficulties. Moreover, the PAUSS captures behavioral manifestations of autistic traits in a more objective and standardized manner. However, unlike self-report tools, it may be less sensitive to subjective experiences or mood states.

## 5. Conclusions

Despite these limitations, the study provides valuable evidence supporting a transdiagnostic perspective on autistic traits in adult psychiatric populations, while also highlighting important differences across diagnostic categories. Social and communication impairments appear to play a central role in distinguishing psychotic spectrum disorders from mood disorders. Future research should aim to replicate these findings in larger, longitudinal samples and to explore the clinical utility of assessing autistic traits to improve diagnostic precision and inform personalized treatment approaches. Furthermore, identifying autistic traits within psychiatric populations is important for understanding heterogeneity in treatment response, particularly to pharmacological interventions. Autistic traits may influence symptom presentation, cognitive style, and underlying neurobiology, thereby contributing to variability in the efficacy and tolerability of medications [72,73,74]. For example, individuals with higher levels of autistic traits may show differential sensitivity to side effects, distinct response trajectories, or reduced benefit from standard treatments targeting social or affective symptoms. Incorporating the assessment of autistic traits into clinical and research settings can therefore support a more stratified approach, helping to identify subgroups of patients who may require tailored pharmacological strategies and improving overall treatment personalization.

## Figures and Tables

**Table 1:** Group differences in psychopathology and autistic traits across diagnostic groups: raw, age-adjusted, and age plus psychopathology-adjusted analyses.

Outcome	MDD Score	BD Score	PSD Score	Raw Analysis		Age-Adjusted		Age-BPRS-Adjusted	
**BPRS**	26.82 ± 5.77	25.7 ± 5.58	33.14 ± 5.06	Group effect	*p* < 0.001	Group effect	*p* < 0.001	Group effect	-
				MDD vs. BD	*p* = 0.529	MDD vs. BD	*p* = 0.882	MDD vs. BD	-
				PSD vs. BD	*p* < 0.001	PSD vs. BD	*p* < 0.001	PSD vs. BD	-
				PSD vs. MDD	*p* < 0.001	PSD vs. MDD	*p* < 0.001	PSD vs. MDD	-
**PAUSS** **Total**	11 (8–13)	10 (8–13)	17 (13–22)	Group effect	*p* < 0.001	Group effect	*p* < 0.001	Group effect	*p* < 0.001
				MDD vs. BD	*p* = 1.000	MDD vs. BD	*p* = 1.000	MDD vs. BD	*p* = 0.668
				PSD vs. BD	*p* < 0.001	PSD vs. BD	*p* < 0.001	PSD vs. BD	*p* < 0.001
				PSD vs. MDD	*p* < 0.001	PSD vs. MDD	*p* < 0.001	PSD vs. MDD	*p* < 0.001
**PAUSS** **Social**	4 (3–5)	4 (3–5.25)	8.5 (7–11)	Group effect	*p* < 0.001	Group effect	*p* < 0.001	Group effect	*p* < 0.001
				MDD vs. BD	*p* = 1.000	MDD vs. BD	*p* = 0.589	MDD vs. BD	*p* = 0.383
				PSD vs. BD	*p* < 0.001	PSD vs. BD	*p* < 0.001	PSD vs. BD	*p* < 0.001
				PSD vs. MDD	*p* < 0.001	PSD vs. MDD	*p* < 0.001	PSD vs. MDD	*p* < 0.001
**PAUSS Communication**	2 (2–3)	2 (2–3)	5 (4–6.25)	Group effect	*p* < 0.001	Group effect	*p* < 0.001	Group effect	*p* < 0.001
				MDD vs. BD	*p* = 1.000	MDD vs. BD	*p* = 1.000	MDD vs. BD	*p* = 0.866
				PSD vs. BD	*p* < 0.001	PSD vs. BD	*p* < 0.001	PSD vs. BD	*p* < 0.001
				PSD vs. MDD	*p* < 0.001	PSD vs. MDD	*p* < 0.001	PSD vs. MDD	*p* < 0.001
**PAUSS RRB**	4 (3–5)	3 (3–4.25)	4 (3–5)	Group effect	*p* = 0.847	Group effect	*p* = 0.960	Group effect	*p* = 0.275
				MDD vs. BD	*p* = 1.000	MDD vs. BD	*p* = 1.000	MDD vs. BD	*p* = 1.000
				PSD vs. BD	*p* = 1.000	PSD vs. BD	*p* = 1.000	PSD vs. BD	*p* = 0.457
				PSD vs. MDD	*p* = 1.000	PSD vs. MDD	*p* = 1.000	PSD vs. MDD	*p* = 0.387

Values are presented as mean ± standard deviation (SD) for normally distributed variables and as median (interquartile range, IQR) for variables that did not meet assumptions of normality. Normality was assessed within each diagnostic group using the Shapiro–Wilk test. Group differences in the raw analysis were tested using one-way analysis of variance (ANOVA) for normally distributed variables and the Kruskal–Wallis test for non-normally distributed variables. When the overall group effect was statistically significant, post hoc pairwise comparisons were performed using Tukey’s honestly significant difference test following ANOVA or Dunn’s test with Bonferroni correction following Kruskal–Wallis analyses. Pairwise contrasts are reported as comparisons between diagnostic groups (MDD vs. BD, PSD vs. BD, PSD vs. MDD). *p*-values refer to the significance of each contrast after the appropriate multiple-comparison correction. Abbreviations: MDD, major depressive disorder; BD, bipolar disorder; PSD, psychotic spectrum disorder; BPRS, Brief Psychiatric Rating Scale; PAUSS, PANSS Autism Severity Score; RRB, restricted and repetitive behaviors.

## Data Availability

The data presented in this study are available on request from the corresponding author.

## Supplementary Materials

The following supporting information can be downloaded at: https://www.mdpi.com/article/10.3390/jcm15124659/s1, Table S1. Nested multinomial logistic regression models examining the sequential contribution of covariates to the association between PAUSS RRB and diagnostic group (reference: MDD). OR = odds ratio; CI = confidence interval.

## Abbreviations

The following abbreviations are used in this manuscript:

ADI-R	Autism Diagnostic Interview-Revised
ADHD	Attention-Deficit/Hyperactivity Disorder
ADOS	Autism Diagnostic Observation Schedule
ANOVA	Analysis of Variance
AQ	Autism-Spectrum Quotient
ASD	Autism Spectrum Disorder
BAPQ	Broad Autism Phenotype Questionnaire
BD	Bipolar Disorder
BPRS	Brief Psychiatric Rating Scale
CHR-P	Clinical High Risk for Psychosis
CI	Confidence Interval
FEP	First Episode Psychosis
IQR	Interquartile Range
MDD	Major Depressive Disorder
OCD	Obsessive–Compulsive Disorder
OR	Odds Ratio
PANSS	Positive and Negative Syndrome Scale
PAUSS	PANSS Autism Severity Score
PCA	Principal Component Analysis
PERMANOVA	Permutational Multivariate Analysis of Variance
PSD	Psychotic Spectrum Disorder
RAADS-R	Ritvo Autism Asperger Diagnostic Scale-Revised
RRB	Restricted and Repetitive Behaviors
SD	Standard Deviation
SRS	Social Responsiveness Scale
VIF	Variance Inflation Factor
