# Supplementary material for: Autistic Trait Profiles Across Mood and Psychotic Spectrum Disorders: A Transdiagnostic Outpatient Study
Source: J Clin Med. 2026 Jun 16;15(12):4659. doi: 10.3390/jcm15124659 (PMC13301547; doi:10.3390/jcm15124659)
Supplement: Supplementary file 1 [file jcm-15-04659-s001.zip › jcm-4321505-supplementary.pdf]

*Article*

# Autistic trait profiles across mood and psychotic spectrum disorders: a transdiagnostic outpatient study

**Table S1.** Nested multinomial logistic regression models examining the sequential contribution of covariates to the association between PAUSS RRB and diagnostic group (reference: MDD). OR = odds ratio; CI = confidence interval.

| Model | Reference group: MDD | OR | 95% CI | *p* value |
|---|---|---|---|---|
| Model 1: RRB only | BD | 0.95 | 0.72–1.27 | 0.744 |
| Model 1: RRB only | PSD | 1.04 | 0.77–1.41 | 0.793 |
| Model 2: + age | BD | 0.97 | 0.73–1.29 | 0.823 |
| Model 2: + age | PSD | 1.02 | 0.75–1.40 | 0.887 |
| Model 3: + age + sex | BD | 0.97 | 0.73–1.29 | 0.818 |
| Model 3: + age + sex | PSD | 0.99 | 0.72–1.36 | 0.945 |
| Model 4: + age + sex + BPRS | BD | 1.02 | 0.75–1.39 | 0.895 |
| Model 4: + age + sex + BPRS | PSD | 0.77 | 0.53–1.11 | 0.156 |
| Model 5: full model | BD | 0.81 | 0.53–1.22 | 0.311 |
| Model 5: full model | PSD | 0.25 | 0.12–0.49 | <0.001 |
